# High rate of transmission in a pulmonary tuberculosis outbreak in a primary school, north-eastern Italy, 2019

**DOI:** 10.2807/1560-7917.ES.2019.24.24.1900332

**Published:** 2019-06-13

**Authors:** Sandro Cinquetti, Maria Dalmanzio, Elisa Ros, Davide Gentili, Mauro Ramigni, Adriano Grossi, Xanthi D Andrianou, Leonardo Ermanno La Torre, Roberto Rigoli, Pier Giorgio Scotton, Angela Taraschi, Vincenzo Baldo, Giuseppina Napoletano, Francesca Russo, Patrizio Pezzotti, Giovanni Rezza, Antonietta Filia

**Affiliations:** 1 Public Health Office , Local Health Unit 2 Marca Trevigiana, Treviso, Italy; 2 Department of Medical, Surgical and Experimental Sciences, University of Sassari, Sassari, Italy; 3 Epidemiology Office, Local Health Unit 2 Marca Trevigiana, Treviso, Italy; 4 University Cattolica del Sacro Cuore, Rome, Italy; 5 Department of Infectious Diseases, National Health Institute (Istituto Superiore di Sanità), Rome, Italy; 6 European Programme for Intervention Epidemiology Training (EPIET), European Centre for Disease Prevention and Control (ECDC), Stockholm, Sweden; 7 Department of Radiology, Oderzo Hospital, Local Health Unit 2 Marca Trevigiana, Treviso, Italy; 8 Department of Microbiology, Treviso Hospital, Local Health Unit 2 Marca Trevigiana, Treviso, Italy; 9 Department of Infectious Diseases, Treviso Hospital, Local Health Unit 2 Marca Trevigiana, Treviso, Italy; 10 Department of Pediatrics, Oderzo Hospital, Local Health Unit 2 Marca Trevigiana, Treviso, Italy; 11 Hygiene and Public Health Unit, University of Padua, Padua, Italy; 12 Prevention Department, Veneto Regional Health Authority, Venice, Italy

**Keywords:** tuberculosis, school outbreak, contact investigation, source-finding investigation, children, health questionnaire, Italy

## Abstract

Italy is a low-incidence country for tuberculosis (TB). We describe a TB outbreak in a primary school in north-eastern Italy, involving 10 cases of active pulmonary disease and 42 cases of latent infection. The index case was detected in March 2019, while the primary case, an Italian-born schoolteacher, was likely infectious since January 2018. Administration of a pre-employment health questionnaire to school staff with sustained contact with children should be considered in low-incidence countries.

Italy has low tuberculosis (TB) incidence, with a notification rate of 6.5 cases per 100,000 in 2017 [1,2], but outbreaks are occasionally described [3-7]. We report a large TB outbreak at a primary school in north-eastern Italy.

## Description of index case and source finding investigation

On 5 March 2019, a case of childhood pulmonary TB was reported to the local health authority (LHA) in a town with a population of approximately 11,000 in the Veneto region (regional TB incidence of 6.2/100,000 in 2017).

The school-aged child had been admitted to hospital for a diagnostic workup because of a 5-week history of fever, asthenia, productive cough, weight loss and diffuse peripheral lymphadenopathy. Prior to admission, the child had been diagnosed with influenza and acute frontal sinusitis, and had received antimicrobials (amoxicillin/clavulanic acid) for 10 days. On admission, the child underwent tuberculin skin testing (TST, Mantoux method: positive, 30 mm), a chest X-ray (CXR) and chest computerised tomography (CT), and a presumptive diagnosis of TB was made. Gastric aspirate (GA) smear microscopy (three samples) was negative. Standard treatment for TB was started.

A source finding investigation was initiated immediately, using the ‘progressive, concentric circles’ (or ‘stone-in-the-pond’) approach [8], by screening household contacts, primary school classmates, schoolteachers and other close contacts of the child, defined as contacts with a cumulative exposure of 8 hours in a small space equivalent to a domestic space [9]. Screening was performed by clinical evaluation, TST and/or interferon gamma release assay (IGRA) testing (in case of previous vaccination or other conditions such as pregnancy, immunosuppression), and/or CXR, according to national guidelines [9]. A positive TST was defined as induration > 5 mm. Persons with a positive TST underwent IGRA testing for confirmation, and CXR to exclude pulmonary active disease. Contacts with CXR abnormalities compatible with TB underwent further diagnostic evaluation, including CT, smear microscopy and/or PCR (sputum or gastric aspirate specimens) and culture if appropriate. TST-positive children were also evaluated clinically by a paediatrician with infectious disease expertise.

As shown in Figure, a total of 35 close contacts of the child, including 22 classmates (class A), eight household contacts, and five teachers, were tested in the source finding investigation.

**Figure:**
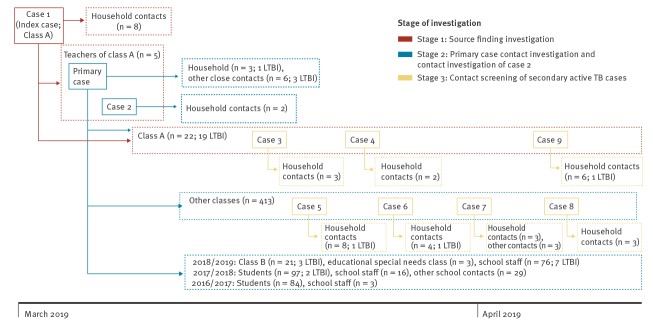
Timeline of the source finding and primary case contact investigations during a school tuberculosis outbreak, north-eastern Italy, 2019

LTBI: latent tuberculosis infection; TB: tuberculosis.

The arrows do not indicate transmission but they connect each active TB case with the contacts screened around the case. The dashed boxes indicate the grouping of contacts and in each box we indicate the number of contacts screened in the group and the number of LTBI found. For each of the active cases, boxes with solid lines are used. The colours indicate the three different stages of the outbreak investigation.

No cases of active TB nor latent tuberculosis infection (LTBI) were found among household contacts of the index case. One of the five schoolteachers, Italian-born, had a positive TST. This teacher reported a non–productive cough since 1 month and no other risk factors. CXR and PCR confirmed active pulmonary TB. Another teacher, despite a negative TST, appeared clinically unwell and reported cough, weight loss and asthenia since 9 months. This teacher therefore underwent further diagnostic evaluation, was found to have extensive cavitary TB, positive microscopy and culture, and was presumed to be the primary case. Drug susceptibility testing showed no resistance to all first-line antibiotics.

All 22 classmates of the index case (case 1) (class A) had a positive TST/IGRA. Four of six children with suspicious CXR findings, all asymptomatic, underwent CT to exclude active disease. Three were diagnosed with active pulmonary TB (Table 1, cases 3, 4 and 9). Gastric aspirate smear microscopy was negative in all three cases, two were culture-positive.

**Table 1:** Characteristics of active tuberculosis cases identified during a primary school outbreak, north-eastern Italy, 2019 (n = 10)

Case	Description	Class	Age (years)	TB site	Symptoms	Chest X-ray	CT scan	Microscopy	PCR	Culture
Primary case	Teacher	A-B	≥ 50	Pulmonary	Persistent productive cough, weight loss	Cavitary TB	Cavitary TB, bilateral tree-in-bud	Positive	Positive	Confirmed
Case 1 (index case)	Student	A	≤ 10	Pulmonary	Fever, persistent and productive cough, asthenia, anorexia	Bronchial wall thickening, hilar enlargement	Multiple enlarged liquefactive hilar lymph nodes	Negative	Negative	Pending
Case 2	Teacher	A-B	≥ 50	Pulmonary	Non-productive cough, (< 1 month)	Minute opacity	None	Negative	Positive (very low)	Pending
Case 3	Student	A	≤ 10	Pulmonary	Asymptomatic	Hilar adenopathy	Pulmonary nodules	Negative	Negative	Confirmed
Case 4	Student	A	≤ 10	Pulmonary	Asymptomatic	Hilar enlargement	Pulmonary nodule, ipsilateral hilar adenopathy	Negative	Negative	Confirmed
Case 5	Student	C	≤ 10	Pulmonary	Asymptomatic	Parenchymal infiltrate, hilar enlargement	None	Positive	Positive (very low)	Confirmed
Case 6	Student	D	≤ 10	Pulmonary	Asymptomatic	Hilar adenopathy	Small patches of lung thickening, multiple enlarged liquefactive hilar lymph nodes	Positive	Positive (very low)	Confirmed
Case 7	Student	E	≤ 10	Pulmonary	Asymptomatic	Hilar adenopathy	Pulmonary nodule, ipsilateral hilar adenopathy	Negative	Positive (very low)	Confirmed
Case 8	Student	C	≤ 10	Pulmonary	Asymptomatic	Hilar adenopathy	Subpleural lung spots, hilar adenopathy	Negative	Negative	Pending
Case 9	Student	A	≤ 10	Pulmonary	Asymptomatic	Parenchymal infiltrate	Pulmonary nodule, multiple hilar lymph nodes	Negative	Negative	Pending

CT: computerized tomography; TB: tuberculosis

## Contact investigation around the primary case

The primary case, Italian-born, was interviewed by experienced staff of the LHA, and reported a persistent cough since June 2018, for which medical attention was sought on two occasions. The cough had been ascribed to smoking and chronic bronchitis, and two antibiotic courses with amoxicillin/clavulanic acid had been completed, 4 months and 1 month prior to diagnosis of TB. The primary case reported a previous positive TST as a child, but had not received preventive treatment.

The primary case taught class A, attended by the index case. The school was a two-story building. Students of class A, all screened in the previous step of the investigation, were considered close contacts, together with three household contacts and six friends/neighbours. One household contact, two neighbours and one friend were TST-positive, none had active TB.

Besides teaching class A, the primary case reported spending 6 hours per week leading workshops for classes A and B, and taught students with special educational needs. The investigation was therefore extended (Figure): three of 21 class B students were TST-positive, all had a normal CXR (Table 2).

**Table 2:** Attack rates for latent and active tuberculosis among screened contacts of the primary case in a school outbreak, north-eastern Italy, 2019 (n = 691)

Contacts of primary case screened	Number at risk	Number screened	LTBI			Active			Infected		
			n	%	(95% CI)	n	%	(95% CI)	n	%	(95% CI)
**School contacts**											
**Students and staff of school (school year 2018/19)**											
Class A^a^	23	23	19	82.6	(61–95)	4	17.4	(5–39)	23	100.0	(85–100)
Class B^b^	21	21	3	14.3	(3–36)	0	0.0	(0–16)	3	14.3	(3–36)
Students with special needs	3	3	0	0.0	(0–71)	0	0.0	(0–71)	0	0.0	(0–71)
Other classes^c^	413	413	7	1.7	(1–3)	4	1.0	(0–2)	11	2.7	(1–5)
School staff	80	80	7	8.8	(4–17)	1	1.3	(0–7)	8	10.0	(4–19)
*Subtotal*	*540*	*540*	*36*	*6.7*	*(5–9)*	*9*	*1.7*	*(1–3)*	*45*	*8.3*	*(6–11)*
**Previous students and staff of school (school year 2017/18)**											
School graduates 2018	99	97	2	2.1	(0–7)	0	0.0	(0–4 )	2	2.1	(0–7)
School staff	18	16	0	0.0	(0–21)	0	0.0	(0–21)	0	0.0	(0–21)
Other contacts	29	29	0	0.0	(0–12)	0	0.0	(0–12)	0	0.0	(0–12)
*Subtotal*	*146*	*142*	*2*	*1.4*	*(0–5)*	*0*	*0.0*	*(0–3)*	*2*	*1.4*	*(0–5)*
**Other close contacts**											
Household contacts	3	3	1	33.3	(1–91)	0	0.0	(0–71)	1	33.3	(1–91)
Friends, neighbours	6	6	3	50.0	(12–88)	0	0.0	(0–46)	3	50.0	(12–88)
*Subtotal*	*9*	*9*	*4*	*44.4*	*(14–79)*	*0*	*0.0*	*(0–34)*	*4*	*44.4*	*(14–79)*
**Total**	**695**	**691**	**42**	**6.1**	**(4–8)**	**9**	**1.3**	**(1–2)**	**51**	**7.4**	**(6–10)**

CI: confidence interval; LTBI: latent tuberculosis infection.

^a^ Including the index case and 22 classmates. Two children were previously vaccinated.

^b^ Including two children born in high-incidence countries. One vaccinated and one unvaccinated.

^c^ Including 14 children born in high-incidence countries, one Italian-born child residing with a foreign-born parent from a high-incidence country and 15 foreign-born vaccinated children.

In view of these findings, the extent of disease and duration of symptoms of the primary case, a decision was made to screen the entire school population (Figure). Four additional active TB cases were identified (Table 1 and Table 2). The investigation was therefore further extended, first to students and staff who had attended or worked in the school in the previous school year (2017/2018), and subsequently to those of school year 2016/2017. Of 97 students and 16 school staff of school year 2017/2018 tested (who no longer attended or worked in the school), two were TST-positive (both had negative CXR), while none of the students and staff of 2016/2017 were TST-positive (Figure; Table 2).

In total, 10 cases of active TB disease were identified, seven were female and three were male. Two cases were teachers (one of whom was the primary case) and eight were students (four of class A and four attending other classes) (Figure; Table 1). Strain testing is currently ongoing. In addition, 31 children, seven teachers, and four close contacts of the primary case were diagnosed with LTBI and were prescribed preventive treatment with isoniazid (Table 2). Two of eight children with pulmonary TB and eight of 31 children with LTBI were either foreign-born or resided in households with foreign-born parents (from high incidence countries). None of the foreign-born parents or other household contacts of these children reported a TB history. Table 2 shows attack rates observed in the outbreak. As shown in the table, 100% of children attending class A were infected.

## Discussion

To our knowledge, this is one of the largest TB school outbreaks detected in Italy in the last 10 years. All cases are considered to have been infected by one of the schoolteachers of class A (primary case), who was Italian-born and had probably been infected as a child when TB incidence in Italy was much higher than it is currently. Molecular typing results will allow us to definitely determine if the cases are all linked. TST results of students attending the school in previous school years suggest that the primary case was infectious since January 2018.

It is unlikely that the second schoolteacher with pulmonary TB was a source of infection in this outbreak as this teacher had a non-productive cough and negative sputum microscopy, and was symptomatic since only 1 month. Likewise, considering that young children are usually not infectious, we doubt that any of them transmitted the infection. Although we consider it improbable, we cannot exclude with absolute certainty that the children with LTBI residing in households with foreign-born parents from higher incidence countries may have been previously infected during visits to these countries. Considering the schoolteacher’s degree of infectivity and duration of symptoms, we expect molecular investigation results to confirm that both children with active TB residing in foreign born families were infected at the school.

The major factor that contributed to this outbreak was delayed diagnosis of the primary case, leading to prolonged exposure of school children and other close contacts, and extensive transmission of the disease. Delayed diagnosis of TB is frequent in outbreaks in low-incidence countries and such a delay occurred also with the index case [4,10,11]. In a recent Italian study, delayed diagnosis of TB occurred more frequently in Italian-born compared with foreign-born cases [12]. Increased awareness of TB is warranted even in low-incidence countries, especially among primary care physicians, and TB should be included in the differential diagnosis of persistent cough, particularly in presence of other symptoms such as unintentional weight loss, fatigue, fever and night sweats.

The contact investigation was conducted by experienced staff of the local public health department and almost all (99.4%) contacts eligible for screening were tested. Effective communication, including face-to-face meetings, with parents, school staff and the public was key in building trust and achieving a high rate of participation. Only one person of 695 did not accept to be tested. The remaining three who were not tested during the school contact investigation had moved to other Italian regions: two have been traced and were tested locally (results pending).

The estimated attack rate in this outbreak was very high for close school contacts. Nineteen of 23 children (82.6%) in class A were diagnosed with LTBI and four of 23 (17.4%) with active pulmonary TB. On the other hand, if we consider all screened contacts, the overall attack rate, seems to be slightly lower compared with other documented outbreaks in Italian schools in the last 10 years [3,4] or in other congregate children settings [13]. This may be due to the limited number of close contacts of the primary case but also to the fact that screening was extended to other-than-close contacts. Contacts with a negative TST at initial investigation will be tested again 10 weeks after the last contact with the primary case.

Currently in Italy, health assessments of primary school employees are not regulated by national law. These assessments are performed mainly from an occupational health perspective that aims to protect employees rather than from a public health perspective that aims to avoid transmission. As already done within the child care system in Sweden [14] and the United States (e.g. in California, at all levels of schooling), the administration of a health questionnaire to school staff who have sustained contact with children should be considered in an effort to identify persons at risk for TB infection (e.g. close contact of someone with infectious TB during lifetime, history of positive TST, birth in a country with an elevated TB incidence) [4,14,15]. The questionnaire could be administered at the stage of employment, to school staff working with children (including staff of nursery schools and child care centres), in order to identify those with risk factors for TB to whom targeted testing (with a TST, IGRA and/or CXR) could be proposed. Based on the screening result, occupational health services staff could determine the need for further evaluation and/or preventive treatment, and decide on the frequency of repeat risk assessments. As highlighted by other authors, besides identifying persons to be screened, the use of such a questionnaire would also provide an opportunity to increase TB awareness among school staff [14].

Improved timeliness of diagnosis of TB is essential to reduce the risk for outbreaks, and timely and efficient investigation of TB outbreaks is fundamental to ensure early detection of TB infection. A recent European Centre for Disease Prevention and Control (ECDC) assessment suggests that information on TB incidents affecting children in congregate settings is not systematically collected and analysed although this would be useful to assess that adequate responses are in place and to share lessons learned in order to improve future source and contact investigations [8].

## Ethical statement

This investigation was part of a routine public health response to a school outbreak and did not require ethics committee approval.
